# Supplementary material for: HIPPO signaling resolves embryonic cell fate conflicts during establishment of pluripotency in vivo
Source: eLife. 2018 Dec 11;7:e42298. doi: 10.7554/eLife.42298 (PMC6289571; doi:10.7554/eLife.42298)
Supplement: Supplementary file 1. [file elife-42298-supp1.docx]

**Supplementary File 1. Summary of embryos recovered from *Wwtr1;Yap1* germline null females**

| Maternal Genotype: *Zp3Cre/+ ; Wwtr1^fl^/Wwtr1^fl^ ; Yap1^fl^/Yap1^fl^*  Paternal Genotype: *Wwtr1^Δ^/+ ; Yap1 ^Δ^/+* | |
| --- | --- |
| Embryo Genotypes (E3.25) – 43 embryos | Number observed (% of embryos) |
| *Wwtr1^Δ^/Wwtr1^Δ^ ; Yap1 ^Δ^/Yap1 ^Δ^* | 4 (9.3%) |
| *Wwtr ^Δ^/Wwtr1 ^Δ^ ; Yap1^Δ^/+* | 11 (25.6%) |
| *Wwtr ^Δ^/+; Yap1^Δ^/Yap1^Δ^ ;* | 13 (30.2%) |
| *Wwtr1^Δ^/+ ; Yap1 ^Δ^/+* | 15 (34.9%) |
| Embryo Genotypes (E3.75) – 60 embryos | Number observed (% of embryos) |
| *Wwtr1^Δ^/Wwtr1^Δ^ ; Yap1 ^Δ^/Yap1 ^Δ^* | 17 (28.3%) |
| *Wwtr ^Δ^/Wwtr1 ^Δ^ ; Yap1^Δ^/+* | 17 (28.3%) |
| *Wwtr ^Δ^/+; Yap1^Δ^/Yap1^Δ^ ;* | 14 (23.3%) |
| *Wwtr1^Δ^/+ ; Yap1 ^Δ^/+* | 12 (20.0%) |
| Embryo Genotypes (All) – 103 embryos | Number observed (% of embryos) |
| *Wwtr1^Δ^/Wwtr1^Δ^ ; Yap1 ^Δ^/Yap1 ^Δ^* | 21 (20.4%) |
| *Wwtr ^Δ^/Wwtr1 ^Δ^ ; Yap1^Δ^/+* | 28 (27.2%) |
| *Wwtr ^Δ^/+; Yap1^Δ^/Yap1^Δ^ ;* | 27 (26.2%) |
| *Wwtr1^Δ^/+ ; Yap1 ^Δ^/+* | 27 (26.2%) |
