# Supplementary material for: HIPPO signaling resolves embryonic cell fate conflicts during establishment of pluripotency in vivo
Source: eLife. 2018 Dec 11;7:e42298. doi: 10.7554/eLife.42298 (PMC6289571; doi:10.7554/eLife.42298)
Supplement: Supplementary file 2. [file elife-42298-supp2.docx]

Supplementary File 2: Mean and standard deviation of cell counts for every experimental treatment

| Figure 1 and S1 | INSIDE | OUTSIDE | TOTAL | INSIDE/OUTSIDE ratio |
| --- | --- | --- | --- | --- |
| Control (n = 17) | 14.6 +/- 4.2 | 23.0 +/- 7.1 | 37.7 +/- 10.4 | 0.66 +/- 0.14 |
| ROCK-inhibitor treated (n = 23) | 10.8 +/- 4.7 | 24.6 +/- 5.3 | 35.3 +/- 8.8 | 0.44 +/- 0.14 |
| Yap1CA/GFP (n = 10) | 18.4 +/- 7.3 | 36.6 +/- 8.9 | 55.0 +/- 10.3 | 0.54 +/- 0.24 |
| Figures 2 and S3 | INSIDE | OUTSIDE | TOTAL | INSIDE/OUTSIDE ratio |
| GFP Only (n = 9) | 15.6 +/- 3.1 | 44.4 +/- 12.8 | 60.1 +/- 12.9 | 0.39 +/- 0.17 |
| Lats2/GFP (n = 18) | 22.9 +/- 7.3 | 32.7 +/- 11.0 | 55.6 +/- 12.3 | 0.79 +/- 0.39 |
| Lats2KD/GFP (n = 7) | 17.4 +/- 4.0 | 51.4 +/- 8.8 | 68.9 +/- 12.2 | 0.34 +/- 0.05 |
| Yap1CA/Lats2/GFP (n = 9) | 25.4 +/- 7.0 | 39.1 +/- 10.7 | 64.6 +/- 9.5 | 0.71 +/- 0.29 |
| Figure 3 | INSIDE | OUTSIDE | TOTAL | INSIDE/OUTSIDE ratio |
| *Sox2* m null Lats2/GFP (n = 5) | 23 +/- 4.6 | 32.8 +/- 8.8 | 55.8 +/- 12.4 | 0.72 +/- 0.15 |
| *Sox2* mz null Lats2/GFP (n = 5) | 19.8 +/- 3.3 | 37.4 +/- 14.6 | 57.2 +/- 13.6 | 0.58 +/- 0.19 |
| Figure 4 | INSIDE | OUTSIDE | TOTAL | INSIDE/OUTSIDE ratio |
| GFP Only (n = 18) | 5.8 +/- 4.9 | 14.8 +/- 3.0 | 20.8 +/- 6.4 | 0.38 +/- 0.31 |
| Lats2/GFP (n = 58) | 4.2 +/- 3.8 | 13.4 +/- 3.5 | 17.2 +/- 6.3 | 0.31 +/- 0.25 |
| Figure 5 | INSIDE | OUTSIDE | TOTAL | INSIDE/OUTSIDE ratio |
| wild type E3.25 (n = 3) | 10.6 +/- 1.5 | 16.6 +/- 2.1 | 27.7 +/- 1.2 | 0.65 +/- 0.17 |
| *Wwtr1* +/-;*Yap1* +/- E3.25 (n = 15) | 9.1 +/- 3.0 | 13.6 +/- 3.6 | 22.6 +/- 5.4 | 0.69 +/- 0.23 |
| *Wwtr1* +/-;*Yap1* -/- or *Wwtr1* -/-;*Yap1* +/- E3.25 (n = 24) | 11.0 +/- 3.2 | 14.7 +/- 3.1 | 25.6 +/- 5.2 | 0.77 +/- 0.22 |
| *Wwtr1* -/-;*Yap1* -/- E3.25 (n = 4) | 12.0 +/- 4.2 | 13.5 +/- 4.4 | 25.4 +/- 4.0 | 0.98 +/- 0.51 |
| Figures 6 and S6 | INSIDE | OUTSIDE | TOTAL | INSIDE/OUTSIDE ratio |
| wild type E3.75 (n = 8) | 16.5 +/- 1.8 | 41.8 +/- 6.8 | 58.3 +/- 7.0 | 0.29 +/- 0.04 |
| *Wwtr1* +/-;*Yap1* +/- E3.75 (n = 8) | 17.6 +/- 5.0 | 31.4 +/- 13.6 | 50.3 +/- 13.9 | 0.36 +/- 0.11 |
| *Wwtr1* +/-;*Yap1* -/- or *Wwtr1* -/-;*Yap1* +/- E3.75 (n = 24) | 25.4 +/- 5.3 | 17.5 +/- 4.0 | 42.9 +/- 7.3 | 0.59 +/- 0.07 |
| *Wwtr1* -/-;*Yap1* -/- E3.75 (n = 13) | 20.8 +/- 2.96 | 15.2 +/- 3.6 | 36.0 +/- 6.2 | 0.58 +/- 0.04 |
