# Supplementary material for: HIPPO signaling resolves embryonic cell fate conflicts during establishment of pluripotency in vivo
Source: eLife. 2018 Dec 11;7:e42298. doi: 10.7554/eLife.42298 (PMC6289571; doi:10.7554/eLife.42298)
Supplement: Supplementary file 3. [file elife-42298-supp3.docx]

**Supplementary File 3. Allele-specific primers used for determining embryo and mouse genotypes**

| **Allele Name** | **Primer Name** | **Primer Sequence** | **Reference** |
| --- | --- | --- | --- |
| *129-Alpl^tm(cre)Nagy^* | Cre-F | ATCCGAAAAGAAAACGTTGA | (Lomelí et al., 2000) |
|  | Cre-R | ATCCAGGTTACGGATATAGT |  |
| *Sox2^tm1.1Lan^* | SOX2 FL/WT R | TGGAATCAGGCTGCCGAGAATCC | (Smith et al., 2009) |
|  | SOX2 F | TCGTTCTGGCAACAAGTGCTAAAGC |  |
|  | SOX2 KO R | AGTACTTTGCTGCCTCTTTAA |  |
| *Tg(Zp3-cre)93Knw* | oIMR1084 | GCGGTCTGGCAGTAAAAACTATC | (de Vries et al., 2000) |
|  | oIMR1085 | GTGAAACAGCATTGCTGTCACTT |  |
| *Wwtr1^tm1.1Eno^* | TAZ FL/WT F | GGCTTGTGACAAAGAACCTGGGGCTATCTGAG | (Xin et al., 2013) |
|  | TAZ FL/WT R | CCCACAGTTAAATGCTTCTCCCAAGACTGGG |  |
|  | TAZ KO FS | TGACAAAGAACCTGGGGCTA |  |
|  | TAZ KO RS | AACTGCTAACGTCTCCTGCC |  |
| *Yap^tm1.1Eno^* | YAP F | ACATGTAGGTCTGCATGCCAGAGGAGG | (Xin et al., 2011) |
|  | YAP FL/WT R | AGGCTGAGACAGGAGGATCTCTGTGAG |  |
|  | YAP KO R | TGGTTGAGACAGCGTGCACTATGGAG |  |
